# A Unique Case of Radiologically Isolated Syndrome Diagnosed During a Follow-up of Cytomegalovirus Meningoencephalitis

**DOI:** 10.7759/cureus.22191

**Published:** 2022-02-14

**Authors:** Zakaria Salimi, Rim Tazi, Asmaa Hazim, Nawal Bouknani, Jehanne Aasfara

**Affiliations:** 1 Department of Neurology, Cheikh Khalifa International University Hospital, Mohammed VI University of Health Sciences, Casablanca, MAR; 2 Neurology, Mohammed VI University of Health Sciences, Casablanca, MAR; 3 Department of Radiology, Cheikh Khalifa International University Hospital, Mohammed VI University of Health Sciences, Casablanca, MAR

**Keywords:** cytomegalovirus, radiologically isolated syndrome, demyelinating diseases, meningoencephalitis, disease modifying therapy

## Abstract

Radiologically isolated syndrome (RIS) refers to an entity in which an MRI of the brain, spine, or both demonstrates incidental white matter lesions that are characteristic of a demyelinating disease in morphology and location. High-risk RIS may require disease-modifying treatment (DMT). A complex interaction among genetic and environmental factors leads to self-reactive immune mechanisms, which are believed to have a pivotal role in the pathogenesis of demyelinating diseases. Viruses are possible triggers to this mechanism. Unlike Epstein-Barr virus (EBV) infection, which is a well-known risk factor for multiple sclerosis (MS), the association between cytomegalovirus (CMV) infection and MS remains uncertain, with some studies indicating a protective effect of CMV on autoimmune diseases. We report a unique case of RIS diagnosed during the follow-up of CMV meningoencephalitis in a patient who presented with generalized seizure onset.

## Introduction

Multiple sclerosis (MS) is a chronic disabling disease of the central nervous system (CNS) that affects the brain, optic nerves, and spinal cord. Its etiopathogenesis is complex and involves genetic susceptibility and environmental exposures, leading to a cascade of events including the activation of the adaptive and innate immune system [1]. Viruses are potential triggers to the disease [2].

Diagnosing MS can be challenging due to its variable clinical features and the lack of specific tests. It combines clinical, imaging, and laboratory evidence. The revised 2017 McDonald criteria have been recommended for this purpose both in clinical practice and research settings [3]. Radiologically isolated syndrome (RIS) involves a condition where individuals with incidental MRI findings consistent with demyelinating disease fulfilling the revised 2017 McDonald criteria for dissemination in space (DIS) [3,4]. The risk assessment of clinical conversion to define MS remains pivotal in targeting patients with the increased benefits of disease-modifying therapy (DMT).

We report an unusual case of cytomegalovirus (CMV) meningoencephalitis with brain MRI findings suggesting demyelinating disease in a 19-year-old immunocompetent female. The diagnosis of RIS was established during the follow-up period.

## Case presentation

**Figure 1:**
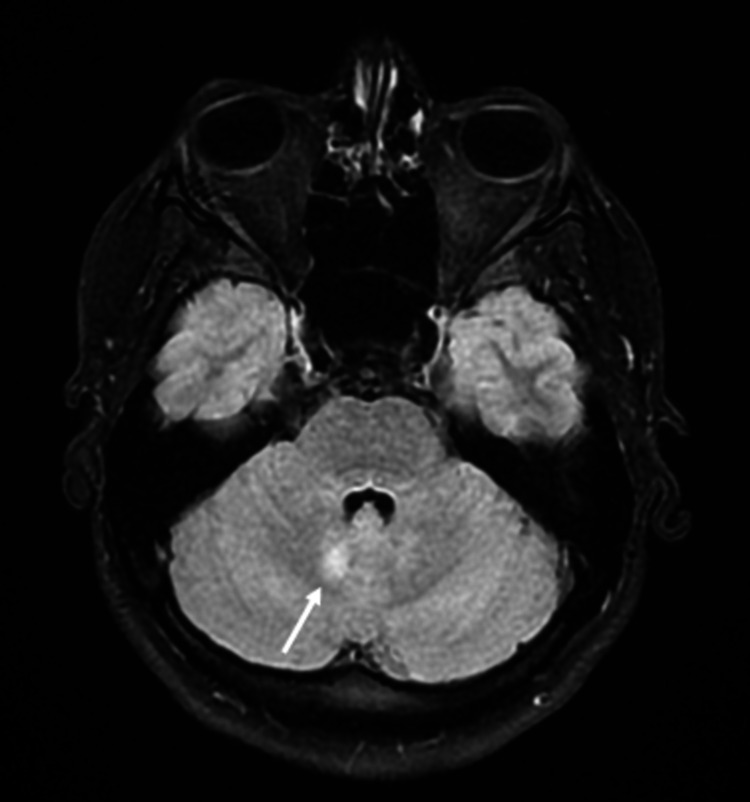
Brain MRI showing hyperintense FLAIR lesion in the cerebellar peduncle (arrow) MRI: magnetic resonance imaging; FLAIR: fluid-attenuated inversion recovery

**Figure 2:**
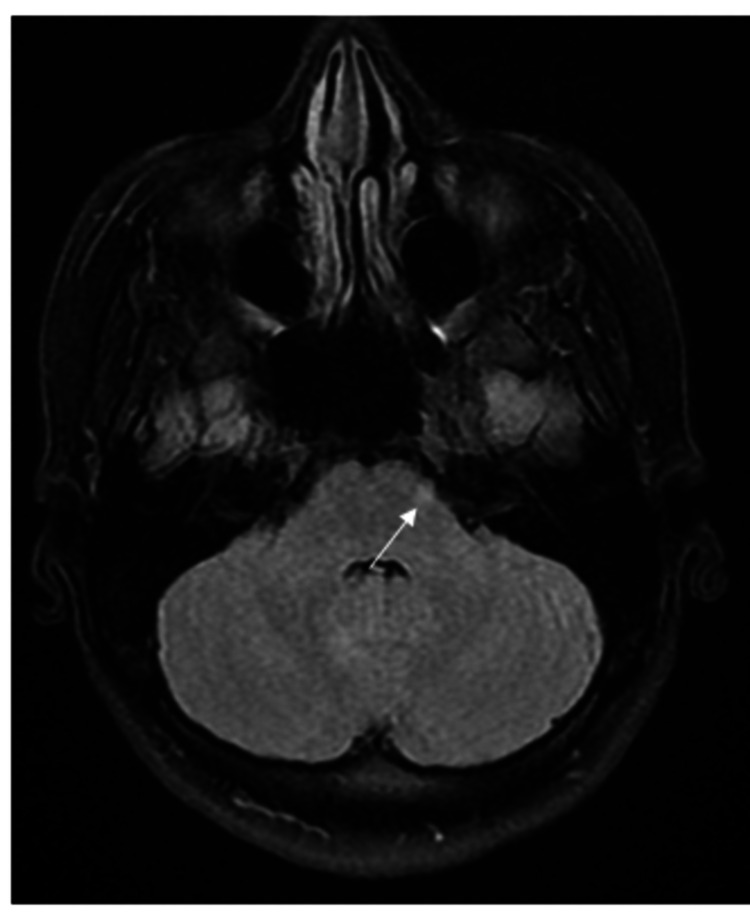
Brain MRI showing hyperintense FLAIR lesion in the rostral lateral pontine area (arrow) MRI: magnetic resonance imaging; FLAIR: fluid-attenuated inversion recovery

**Figure 3:**
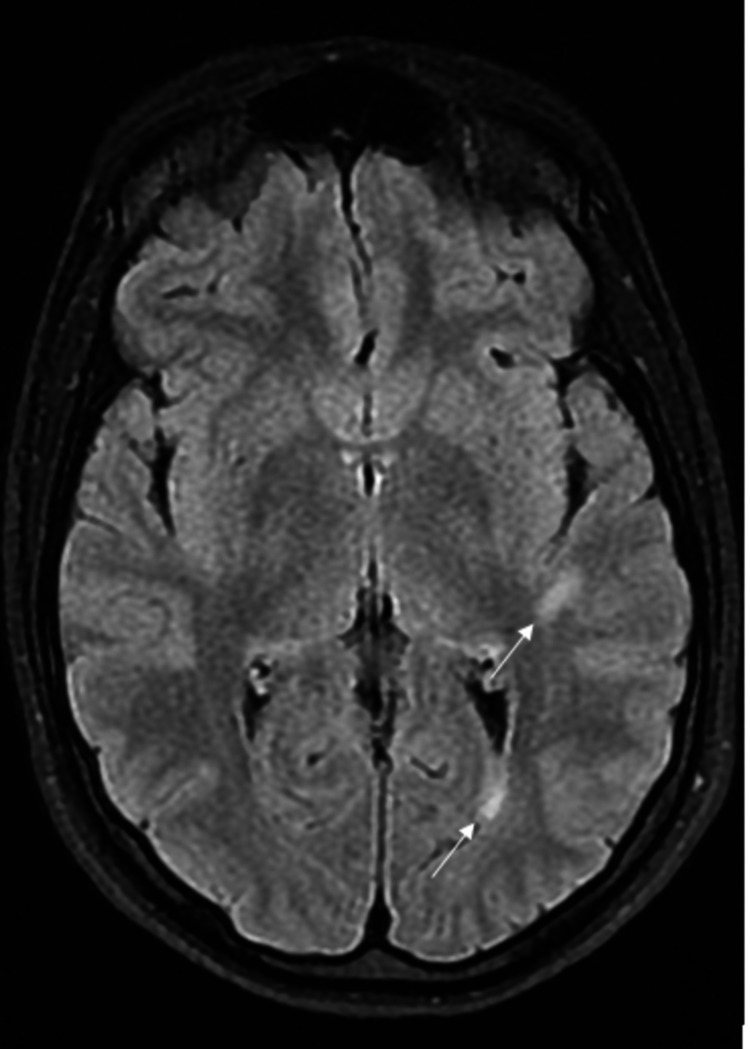
Brain MRI showing periventricular and subcortical hyperintense FLAIR lesions (arrows) MRI: magnetic resonance imaging; FLAIR: fluid-attenuated inversion recovery

A 19-year-old female patient presented with acute headache, nausea, stiff neck, and fever. She was admitted for generalized onset motor seizures. Her body temperature on admission was 38.5 °C. Kernig and Brudzinski's signs were positive with moderate neck rigidity. Neurologic examination found no motor, sensory, visual, or cranial impairment. Laboratory tests showed a sedimentation rate of 13 mm/h, leucocytes of 10.03 x 10^3^/L, and C-reactive protein of 1.19 mg/l. Brain MRI showed four hyperintense fluid-attenuated inversion recovery (FLAIR) lesions in the cerebellar peduncle (Figure 1), rostral lateral pontine area (Figure 2), as well as periventricular and subcortical lesions (Figure 3) with no gadolinium enhancement.

Electroencephalogram (EEG) showed focal epileptic discharges on the left temporal region. Cerebrospinal fluid (CSF) analysis revealed a value of 150 cells/mm^3^ (2% neutrophils, 98% lymphocytes) and normal levels of protein and glucose CSF/serum ratio. CSF culture and microscopy were negative. CMV antigen was detected by PCR in the CSF at a significant rate (3,85 log UI/mL). Antigens of *Cryptococcus neoformans*, herpes simplex virus (HSV), varicella-zoster virus (VZV), and Epstein-Barr virus (EBV) were not found in the CSF. However, CSF oligoclonal bands (OCB) detection was not conducted as meningitis could produce a false positive test. Investigations for underlying immunosuppression, (HIV, hepatitis B and C, and syphilitic serologies) showed no abnormalities up to three months.

The patient’s clinical picture and laboratory and imaging studies were compatible with CMV meningoencephalitis with radiological features suggesting demyelinating disease not fulfilling the MS diagnostic criteria for DIS and dissemination in time (DIT). The patient was started on intravenous ganciclovir 300 mg twice a day for 21 days along with levetiracetam. She had a progressively good recovery.

**Figure 4:**
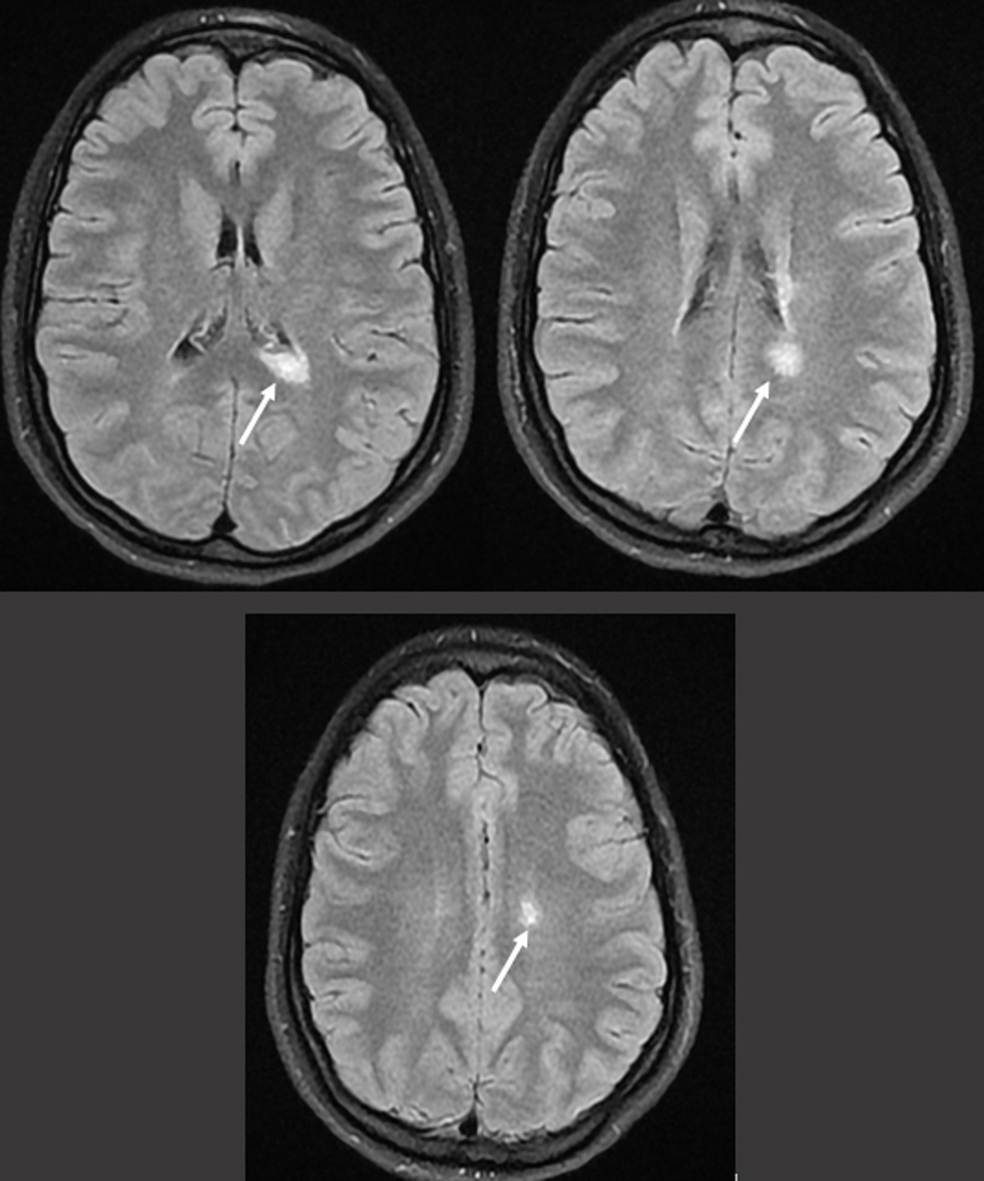
Brain MRI follow-up at six months showing three new hyperintense FLAIR periventricular lesions (arrows) MRI: magnetic resonance imaging; FLAIR: fluid-attenuated inversion recovery

Six months after the initial onset, she presented with focal and generalized onset motor seizures, 20 days after the degression of levetiracetam. Brain MRI showed three new hyperintense FLAIR lesions on the periventricular region and subcortical white matter (Figure 4) with no gadolinium enhancement or abnormal signals on the spinal cord. Focal epileptic discharges were still found on the left temporal region on EEG.

CSF analysis found no hypercellularity, and there were normal levels of glucose and protein in CSF. However, CSF OCBs were detected. Anti-AQP4-IgG and Anti-MOG-IgG antibodies in blood were not found. Biological and radiological tests excluded infectious, toxic, metabolic, or systemic inflammatory diseases. With the criteria for DIS and DIT being fulfilled with ''no better explanation'', a diagnosis of high-risk RIS was established and first-line treatment with injectable immunomodulator was recommended.

## Discussion

RIS refers to a condition where individuals with MRI abnormalities highly suggestive of demyelinating pathology fulfilling the revised 2017 McDonald criteria for DIS but without a clinical history of demyelinating attacks or ongoing neurologic deterioration or other alternative causes of the white matter lesions such as those from vascular, infectious, toxic, and drug-related pathology [5]. In 2009, Okuda et al. proposed diagnostic criteria for RIS, which had been validated among a worldwide cohort [4]. In 2017, the Magnetic Resonance Imaging in MS (MAGNIMS) European group offered a better definition of the imaging criteria for RIS. The MAGNIMS consensus group did not propose a strict recommendation on diagnosing and treating RIS; however, they suggested that identical MRI criteria for DIS and DIT should be used for RIS and MS (Table 1) [5].

**Table 1:** 2017 Mcdonald criteria for the demonstration of dissemination in space and time by MRI MRI: magnetic resonance imaging

Dissemination in space (DIS)	Dissemination in time (DIT)
One or more T2 lesions in at least 2 of these 4 areas:	New T2/gadolinium-enhancing lesion on follow-up MRI
Periventricular	OR simultaneous presence of gadolinium-enhancing and non-enhancing lesions at any time
Juxtacortical	
Infratentorial	
Spinal cord	

Since then, prospective studies have been conducted to assess the risk factors of clinical symptoms among patients with RIS. These studies have speculated that young age, the presence of OCBs on the CSF profile, and infratentorial, spinal, and gadolinium-enhancing lesions were associated with an increased risk, with an estimated 10-year risk of 51% for an acute or progressive demyelinating event [6].

To our knowledge, our patient is the first case of RIS diagnosed along with CMV meningoencephalitis in an immunocompetent individual. In our patient, a follow-up brain MRI established the diagnosis of RIS meeting the criteria for DIS and DIT without clinical event related to the demyelinating disease. Therefore, we underline the relevance of adopting the modified criteria for diagnosing RIS proposed by the MAGMINS European group even though they do not include strict recommendations [5]. In our case, RIS could have been diagnosed at the first MRI even if Barkhof criteria were not fulfilled. A disease-modifying treatment may have prevented the worsening of demyelinating findings.

We presume that seizure onsets reported in our case cannot be related to RIS. Existing knowledge on epilepsy in MS indicates that seizures are rarely the heralding symptom, occurring in patients with high disability levels at the first onset, and more often among secondary progressive MS patients with an elevated proportion of focal seizures [6,7]. These features were not found in our case. In addition, epilepsy in the current context was more likely linked to CMV meningoencephalitis and degression of the anti-epileptic drug. Therefore, even if criteria for DIS and DIT are fulfilled in our case, we cannot confirm a diagnosis of MS as no clinical attack has been reported.

Given the young age of the patient, infratentorial lesions, and OCB in the CSF, RIS in our case should be classified as a high MS risk entity [8] and should therefore be treated with first-line safe DMT [9]. Another particularity in our case was the initial presentation of CMV meningoencephalitis. Many studies have discussed the role of environmental factors and exposures in MS pathogenesis. Unlike EBV, which shows strong incriminating evidence for its role in MS [2], the association between CMV and MS risk is not clear. Some studies have even suggested a protective effect of CMV on autoimmune diseases and MS, possibly by triggering immunomodulatory or evasion mechanisms and consequently decreasing the immune response [10,11].

CMV has been found in demyelinating plaques of immunocompromised MS patients, which was not the case with our patient [12]. CMV infection has also been implicated in the worsening of autoimmune diseases or the progression of acute disseminated encephalomyelitis (ADEM) to clinically definite MS [13]. Animal models have suggested that a "delayed molecular mimicry" mechanism with cross-reactivity between a human CMV peptide and myelin oligodendroglia glycoprotein (MOG) peptide has a role in MS [14]. These findings could explain the worsening of demyelinating MRI lesions at the follow-up in our case, leading to the diagnosis of RIS.

## Conclusions

We reported an unusual case of RIS with CMV meningoencephalitis, and it highlights the value of a follow-up brain MRI in patients with incidental MRI findings suggesting demyelinating disease. We believe that the criteria proposed by the MAGNIMS European group may be highly relevant for establishing an early diagnosis of RIS. The risk assessment of clinical conversion to define MS is critical to targeting patients with the high benefits of DMT. Nevertheless, further studies are required to elucidate a possible link between CMV infection and demyelinating diseases.
